# Cortical glutamate and gamma-aminobutyric acid over the course of a provoked migraine attack, a 7 Tesla magnetic resonance spectroscopy study

**DOI:** 10.1016/j.nicl.2021.102889

**Published:** 2021-11-24

**Authors:** Gerrit L.J. Onderwater, Jannie P. Wijnen, Chloé Najac, Robin M. van Dongen, Itamar Ronen, Andrew Webb, Ronald Zielman, Erik W. van Zwet, Michel D. Ferrari, Hermien E. Kan, Mark C. Kruit, Gisela M. Terwindt

**Affiliations:** a Department of Neurology, Leiden University Medical Center, Leiden, The Netherlands; b Department of Radiology, C.J. Gorter Center for High Field MRI, Leiden University Medical Center, Leiden, The Netherlands; c Department of Radiology, University Medical Center Utrecht, Utrecht, The Netherlands; d Department of Medical Statistics and Bioinformatics, Leiden University Medical Center, Leiden, The Netherlands; e Department of Radiology, Leiden University Medical Center, Leiden, The Netherlands

**Keywords:** Migraine, Glutamate, GABA, Magnetic resonance spectroscopy, Glyceryl trinitrate, CRLB, Cramér-Rao lower bound, CSF, cerebrospinal fluid, FWHM, full width half-maximum, Glx, glutamate and glutamine, GM, grey matter, GTN, glyceryl trinitrate, ^1^H-MRS, proton magnetic resonance spectroscopy, HIT-6, headache impact test, MIDAS, migraine disability assessment scale, SNR, signal to noise ratio, tNAA, total *N*-acetylaspartate, VOI, volume of interest, WM, white matter

## Abstract

Enhanced activity of the glutamatergic system has been linked to migraine pathophysiology. The present study aimed to assess the involvement of the glutamatergic system in the onset of attacks. We provoked attacks by infusion of glyceryl trinitrate (GTN; 0.5 µg/kg/min over 20 min) in 24 female episodic migraineurs without aura and 13 female age-matched healthy controls. Over the course of a single day participants were scanned three times at fixed time slots (baseline before GTN infusion, 90 min and 270 min after start of GTN infusion). Single-volume proton magnetic resonance spectra (^1^H–MRS) were acquired at 7 Tesla from a volume of interest (VOI, 2x2x3 cm) in the visual cortex. We assessed the concentrations of glutamate, its major precursor glutamine, and its product gamma-aminobutyric acid (GABA) over the course of a provoked attack. The preictal state was defined as the period after GTN infusion until the migraine-like headache started, independent of possible experienced premonitory symptoms, and the ictal state was defined as the period with provoked migraine-like headache. Data were analyzed using a linear mixed-effect model for repeated measures. Glutamate and glutamine levels did not change from interictal to the preictal and ictal state. GABA levels increased from interictal towards the preictal state for migraine patients compared with healthy controls. We conclude that high resolution 7T MRS is able to show changes in the glutamatergic system towards a triggered migraine attack, by revealing an increased GABA concentration associated with the onset of a migraine attack.

## Highlights

• 7T MRS separately measured glutamate, glutamine and GABA towards triggered attacks.
• Visual cortex GABA levels increased towards a preictal migraine state.
• Visual cortex glutamate and glutamine levels were stable across migraine states.

## 1 Introduction

Migraine is a brain disorder affecting 15% of the global population (Global Burden of Disease Study 2016 Collaborators, 2017). Attacks are characterized by headache accompanied by nausea, vomiting and/or photo- and phonophobia (migraine without aura) (Headache Classification Committee of the International Headache Society (IHS), 2018). Transient spreading focal neurological symptoms, caused by cortical spreading depolarization (CSD) occur in one-third of patients (migraine with aura) (Launer et al., 1999). A typical migraine attack consists of a preictal (premonitory), ictal (aura and/or headache), and postictal phase (Headache Classification Committee of the International Headache Society (IHS), 2018). The pathophysiological mechanisms behind initiation of attacks are still not fully elucidated.

Migraine attack susceptibility is thought to be related to dysregulation of excitability of the brainstem, deep brain nuclei and cortex, (Bolay, 2012, Cosentino et al., 2014) which may (at least partly) be explained by changes in glutamatergic neurotransmission (Ferrari et al., 2015). Enhanced excitability, possibly caused by glutamate-related changes, may directly increase the susceptibility to develop CSD, or the reactivity of certain brain areas to stimuli, such as photophobia (Boulloche et al., 2010, Ferrari et al., 2015, Maniyar et al., 2014a, Maniyar et al., 2014b). The potential role of glutamate-related changes in migraine is supported by the finding of elevated glutamate levels in cerebrospinal fluid (CSF) of chronic migraine patients, in blood of interictal episodic migraine patients, and in the visual cortex of interictal episodic migraineurs without aura using high-field magnetic resonance spectroscopy (7T-MRS) (van Dongen et al., 2016, Zielman et al., 2017). The likeliness that the visual cortex is not only relevant in migraine with aura, but is also implicated in migraine without aura patients, is further illustrated by functional magnetic resonance imaging (fMRI) studies showing activations in the occipital cortex in migraine without aura patients in the premonitory phase (Maniyar et al., 2014a, Maniyar et al., 2014b, Schulte and May, 2016).

The excitatory neurotransmitter glutamate not only plays an important role in neurotransmission and excitatory-inhibitory balance, together with the inhibitory neurotransmitter gamma-aminobutyric acid (GABA), but also functions in energy and amino acid metabolism (Waagpetersen et al., 2007). Intracellular glutamate is compartmentalized in distinct pools (about 80% neuronal and 20% astrocytic) (Erecinska and Silver, 1990, Waagpetersen et al., 2007). This astrocytic glutamate pool can be formed into the non-neuroactive amino acid glutamine, by glutamine synthase which is exclusively expressed in glial cells. Astrocytic glutamine can subsequently be deamidated and re-formed into glutamate by glutaminase after transferal to glutamatergic or GABAergic neurons (Schousboe et al., 2013, Waagpetersen et al., 2007, Walls et al., 2014). Glutamate can also be formed from and transformed into, α‐ketoglutarate, an intermediate of the tricarboxylic acid (TCA) cycle (Waagpetersen et al., 2007). This neuronal glutamate can enter the TCA cycle, be used as a neurotransmitter (glutamatergic neurons) or be transformed to GABA (GABAergic neurons) (Schousboe et al., 2013, Waagpetersen et al., 2007, Walls et al., 2014). Active transportation of glutamate and GABA into neurons and astrocytes is used to preserve neurotransmission (Schousboe et al., 2013). These components form the glutamatergic system, (Walls et al., 2014) elements of which have been studied *in vivo* using proton magnetic resonance spectroscopy (^1^H-MRS). However, results have been difficult to interpret and are conflicting, likely due to methodological shortcomings (Peek et al., 2020, Reyngoudt et al., 2012, Younis et al., 2017, Zielman et al., 2017). Studies generally reported on the combined MRS-signal of glutamate and glutamine (Glx) (Danbolt, 2001, Waagpetersen et al., 2007). As glutamate is also metabolically intertwined with GABA (Walls et al., 2014), measuring the concentration of each these three metabolites separately might be essential to detect possible conversions between these metabolite pools.

As spontaneous attacks occur unexpectedly and related disability frequently inhibits patients from traveling, or the attack is already in the ictal phase when arriving at the hospital, studying attack initiation is challenging (Ashina et al., 2017). Therefore, experimental migraine models such as intravenous glyceryl trinitrate (GTN) have been used to investigate attack initiation under precisely monitored and regulated conditions (Ashina et al., 2017). Glyceryl trinitrate provocation studies have only rarely been able to provoke aura symptoms and generally provokes migraine-like attacks in over eighty percent of migraine without patients (Ashina et al., 2013). In both GTN provoked and spontaneous attacks patients may experience premonitory symptoms in the preictal phase, and using neuroimaging techniques activation of hypothalamus, occipital cortex and brainstem has been demonstrated (Ferrari et al., 2015, Maniyar et al., 2014a, Maniyar et al., 2014b, Onderwater et al., 2020, Schulte and May, 2016; van Oosterhout et al., 2021, Weiller et al., 1995).

In this study, we aimed to investigate changes in the glutamatergic system in the visual cortex before and during the initial phases of provoked migraine-like attacks in migraine patients without aura and compare these results with healthy controls by using single-volume ^1^H-MRS at 7 Tesla.

## 2 Material & methods

### 2.1 Participants

We included 25 female migraine without aura patients, and 14 age-matched female healthy controls (group matched; by adhering to 5-year age strata). Migraine without aura patients were selected because of the following reasons. Firstly, elevated CSF and blood glutamate levels were found in a review in groups made up for a majority of migraine without aura patients (van Dongen et al., 2016). Secondly, in a 7 Tesla MRS study, interictal migraine patients (migraine with and without aura patients), elevated glutamate levels were detected only in migraine without aura patients (Zielman et al., 2017). Thirdly, the premonitory (preictal) phase in migraine without aura patients during spontaneous and as well as provoked attacks revealed activations in the visual cortex (Maniyar et al., 2014a, Maniyar et al., 2014b, Schulte and May, 2016). Finally, GTN infusion has been shown to only sporadically provoke aura symptoms, even in migraine patients with (hemiplegic) aura, while it generally is able to provoke migraine-like attacks most successfully in migraine without aura patients (Ashina et al., 2017). Participants were recruited from the Leiden University Medical Center Migraine Neuro Analysis (LUMINA) project in which migraineurs and healthy controls from the Dutch population who have agreed to participate in migraine-related scientific research are listed, and also by public advertisement. Migraine without aura was diagnosed in accordance with the International Classification of Headache Disorders (ICHD-3) (Headache Classification Committee of the International Headache Society (IHS), 2018). Participants with migraine without aura were otherwise healthy, and experienced at least one migraine attack per month in the preceding six months and did not have chronic migraine or medication overuse headache (or caffeine overuse headache). Healthy controls were free of any known neurological or psychiatric disorders and did not have any primary or secondary headaches apart from occasional episodic tension-type headache. Furthermore, healthy controls did not report a first degree family member with migraine or trigeminal autonomic cephalalgia. None of the participants used any chronic medication other than oral contraceptives. The study was approved by the ethics committee of the Leiden University Medical Center. All participants provided written informed consent prior to the study.

### 2.2 Study design

**Fig. 1:**
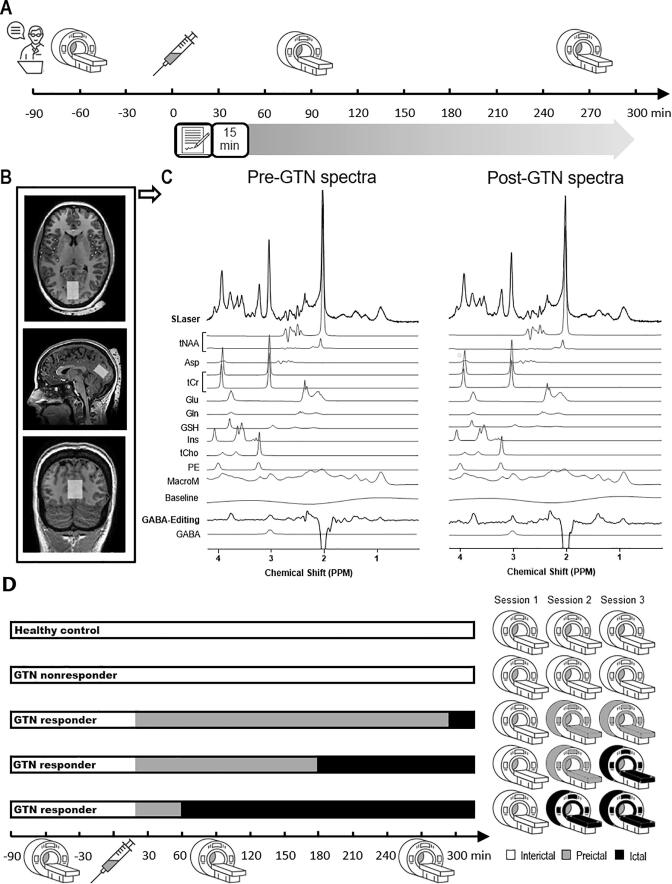
Study design and MRS methodology. (A) Study design with a baseline assessment (neurological examination, headache assessment, MR contra-indications), glyceryl trinitrate infusion (GTN; 0.5 µg/kg/min over 20 min), and MRS scans in combination with blood glucose at baseline, 90 min and 270 min after the start of GTN infusion. (B) T1-weighted transverse, sagittal, and coronal images outlining volume of interest (20 × 20 × 30 mm) positioning in the occipital lobe indicated by the white box. Extracranially the dielectric pad can be seen. (C) Representative examples of pre-GTN and post-GTN acquired SLASER and GABA-edited spectra with separate fitting of the included metabolites in combination with the baseline which includes signal contributions of residual lipids and water. tNAA = total *N*-acetylaspartate (composed of *N*-acetylaspartate and *N*-acetylaspartylglutamate), Asp = aspartate, tCr = total creatine (composed of creatine and phosphocreatine), Glu = glutamate, Gln = glutamine, GSH = glutathione, Ins = myo-inositol, tCho = total choline (composed of glycerophocholine), PE = phosphoethanolamine, MacroM = macromolecules, PPM = parts per million. (D) Possible reactions to GTN for healthy controls, GTN nonresponders and GTN responders, with in white the interictal phase, in grey the preictal phase and in black the ictal (migraine-like headache) phase in the different bars showing the course of the study day. The MR icons representing the three fixed scan sessions (Baseline, GTN-90 and GTN-270) for each example illustrate the applied value for the variable (migraine phase) in the mixed model (white = interictal, grey = preictal, and black = ictal). The bars and corresponding MR icons for healthy controls and GTN nonresponders illustrating these groups are classified as interictal throughout the study day. The top GTN responder bar and corresponding MR icons illustrate migraine-like headache onset after the 3rd scan session thereby the 2nd and 3rd scan session are classified as preictal. The middle GTN responder bar and corresponding MR icons illustrate migraine-like headache onset prior to the 3rd thereby the 2nd and 3rd scan sessions are classified as preictal and ictal, respectively. The bottom GTN responder bar and corresponding MR icons illustrate migraine-like headache onset prior to the 2nd scan session thereby the 2nd and 3rd scan sessions are both classified as ictal.

Prior to participation interested individuals were screened using a standardized telephonic interview in order to assess suitability for participation. Each participant was examined during a single study day, which included a detailed interview and three MRI-scans at fixed time slots. Participants were instructed to abstain from smoking and from consuming any alcoholic or caffeinated beverages for at least eight hours prior to the study day to minimize possible bias. Furthermore, participants refrained from using prophylactic medication for at least four weeks and were attack-free at least three days prior to the investigation. Participants were allowed to eat prior to and during the course of the day. Prior to the first scan session [Baseline] all participants underwent a baseline assessment including a neurological examination and headache assessment. Participants were instructed to keep their eyes closed during the scan sessions. To minimize bias due to eventual diurnal effects, scanning started around 8:30 am. Blood glucose levels were ascertained after each scan session, because glucose may affect glutamate, glutamine and GABA concentrations via the TCA cycle (Hertz, 2013, Lai et al., 2018). After the baseline scan session, participants received an infusion of GTN (0.5 µg/kg/min over 20 min). Afterwards participants were scanned two more times: 90 min after start of GTN infusion [GTN-90] and 270 min after GTN infusion [GTN-270], as shown in Fig. 1A. Participants were asked to refrain from using their acute migraine attack medication until after the final MRS scan to avoid influencing biochemical processes related to the onset of a migraine attack. Participants completed a headache assessment every five minutes during GTN infusion and every 15 min after GTN infusion until the end of the study day five hours later (except for the time during the MRS scan). Participants with migraine kept a headache diary for seven days before and seven days after the study day (healthy controls only kept a headache diary after the study day) and completed questionnaires on migraine characteristics. Furthermore, participants were followed up by a phone call around 3 days after participation to monitor response after GTN infusion and identify late responders and confirm nonresponders.

### 2.3 Migraine-like headache and criteria

Participants were informed that GTN could potentially induce headache, without information regarding the expected onset and course of the headache. Migraine provocation with GTN typically follows a biphasic pattern; it first induces immediate headache in migraine patients as well as healthy controls, after which migraineurs may develop a delayed headache fulfilling the criteria for migraine without aura within 12 h (Ashina et al., 2017). Headache assessments were obtained using a predefined response form including: verbal rating scale (VRS), headache localization, type of pain, associated symptoms, nonheadache (premonitory) symptoms, and adverse events. Despite their similarity with spontaneous attacks, induced attacks need to be referred to as ‘migraine-like headaches’, because by nature they cannot fulfill the criteria for migraine without aura, which require the attack to be spontaneous and last (untreated) at least 4 h (Headache Classification Committee of the International Headache Society (IHS), 2018). Therefore, similarly to previously published provocation studies, we used the following criteria for defining migraine-like attacks, fulfilling either: 1) moderate to severe headache (VRS ≥ 4) fulfilling ICHD-3 criteria C and D for migraine without aura; or 2) headache described as mimicking the patients' usual migraine attack and treated with acute migraine medication (Arngrim et al., 2016, Onderwater et al., 2020).

### 2.4 MRS data acquisition

Participants were examined using ^1^H-MRS on a 7 Tesla MR system (Philips Healthcare, Best, The Netherlands) using a 32 channel receive array and a quadrature transmit coil (Nova Medical, Wilmington, MA, USA) powered by two amplifiers (4 kW each). We optimized the phase setting between the two amplifiers for each subject to generate a local transmit field (B_1_) of 17 µT in the region of interest. A deformable dielectric pad was positioned at the posterior side of the head over the occipital bone (Snaar et al., 2011). An anatomical 3D T_1_-weighted gradient echo image was acquired to ensure accurate planning of the volume of interest (VOI) for MRS (Fig. 1B). Imaging parameters were: field of view: 246 × 246 × 174 mm^3^, resolution 1 × 1 × 1 mm^3^, repetition time (TR)/echo time (TE) = 4.9/2.2 ms.

To measure glutamate, glutamine, and other major metabolites we used a single-volume ^1^H-MRS semi-localized by adiabatic selective refocusing (sLASER) sequence (TR = 5000 ms, TE = 36 ms, spectral width = 4 kHz, 2048 points, 32 averages, acquisition time ≈ 3 min) (Boer et al., 2011). Acquisition was preceded by a variable power and optimized relaxation delays (VAPOR) water suppression sequence (Tkáč et al., 1999). To measure GABA levels, single-volume ^1^H-MRS spectra with J-difference spectral editing (GABA-edited ^1^H-MRS) were obtained using a Mescher-Garwood (MEGA)-sLASER sequence with macromolecule suppression by alternating the offset frequency of the editing pulse symmetrically around GABA (1.5 and 1.9 ppm) (TR = 5000 ms, TE = 74 ms, spectral width = 4 kHz, 2048 points, 64 averages, acquisition time ≈ 6 min) (Andreychenko et al., 2012). Water suppression was achieved via the spectral selectivity of both MEGA pulses and therefore acquisition proceeded without additional water suppression. To optimize editing efficiency, frequency offset corrected inversion (FOCI) refocusing pulses were used with a B_1_ amplitude of 17µT and an inversion band width of 7 kHz (Arteaga de Castro et al., 2013).

Second order static magnetic field (B_0_) shimming on the VOI was performed to ensure a highly homogenous localized B_0_ field. Both ^1^H-MRS spectra were acquired in the same manually planned 30 × 20 × 20 mm^3^ VOI including a non-water-suppressed spectrum, with the transmitter frequency set on the water resonance. ^1^H-MRS spectra were pre-processed with a custom written script in Matlab® (The MathWorks, Inc., Natick, MA, USA) that yielded a weighted average of the individually-phased signals from all 32 receive channels, frequency alignment and eddy current correction.

All participants were included and scanned by one investigator (G.L.J.O.). To avoid bias, clear anatomical landmarks for VOI placement were used. The VOIs were centered along the calcarine fissure, symmetrically covering both hemispheres caudal of the parieto-occipital fissure and including the primary and secondary visual cortices (Brodmann areas 17 and 18; Fig. 1B) (Zielman et al., 2017). After study completion, an independent investigator (R.M.v.D.), blinded to subject status, investigated correct VOI placement.

### 2.5 Data-processing and quality monitoring

To account for differences in water concentration and relaxation times in the absolute quantification of metabolites, we evaluated tissue fractions (grey matter, white matter and CSF) within the VOI, and used these later for absolute quantification (Wang and Li, 1998). Tissue fractions within the VOI were calculated based on the 3D T_1_ images, after applying the Brain Extraction Tool and whole brain segmentation with the Automated Segmentation Tool from FSL (version 5.0.9, FMRIB Software Library, University of Oxford).

The sLASER ^1^H-MRS spectra were analyzed using LCModel (version 6.3–1 K, Stephen Provencher, Inc., Oakville, ON, Canada) (Provencher, 2001). The parameter DKNTMN that controls the node spacing for the spline baseline fitting was set to 1. For an overview of applied LCModel control parameters, see e-table 1, supplementary materials. To fit the spectra, we initially used a simulated basis set generated using FID Appliance (open-source Matlab-based software toolkit) (Simpson et al., 2017), an acquired macromolecular spectrum (with a Double Inversion Recovery sequence) was also added as a model signal to the basis set which in total was composed of 24 metabolites. Only metabolites with Cramér-Rao Lower Bound (CRLB) equal to or lower than 15% in over 50% of all baseline acquisitions were included in the final basis set of 15 metabolites, in order to minimize the risk of overfitting. Eventually, however, this did not affect our main outcomes (e-table 2, supplementary materials). GABA-edited ^1^H-MRS spectra were analyzed with a custom written script in Matlab® which performed fitting of GABA and creatine resonances to Lorentzian line shapes by frequency-domain fitting (Andreychenko et al., 2013).

The ^1^H-MRS spectra were visually inspected by two investigators (G.L.J.O. and J.P.W.) who were blinded for the diagnosis. Spectra showing clear *a priori* determined artifacts, e.g. due to stimulated echoes, inadequate water suppression, or poor shimming, were excluded. The LCModel signal to noise ratio (SNR), defined as the ratio of the maximum in the spectrum minus the averaged baseline divided by twice the root-mean-square of the residuals between 0.2 and 4.2 ppm, was used as a parameter to assess spectral quality (Provencher, 2001). The full width at half-maximum (FWHM) of NAA (*N*-acetylaspartate), which is a measure of the B_0_ homogeneity, was a second quality measure. Finally, the CRLB, expressed as the estimated standard deviation in percentage of the estimated metabolite concentration, was a final quality measure. The custom written Matlab® script used for the analysis of GABA-edited ^1^H-MRS spectra provided the area under the curves of creatine and GABA (corrected for editing efficiency) (Andreychenko et al., 2012). The SNR of creatine, determined as area under the curve of creatine divided by the standard deviation of the noise in a signal-free part of the spectrum (8–10 ppm) and GABA CRLB, determined as described in Cavassila et al. (Cavassila et al., 2001), were used to assess spectral quality. CRLB values smaller than 15% SD on average were considered reliable estimates of the metabolite concentration (e.g. glutamate, GABA, or NAA); if the CRLB of a given metabolite exceeded 15% SD in more than 50% of the cases that metabolite was excluded from further analysis for all cases (Zielman et al., 2017).

### 2.6 Metabolite quantification

Spectral quantification was performed using the unsuppressed water signal obtained from the same VOI (Gasparovic et al., 2006). The relative densities of MR-visible water for grey matter, white matter and CSF were assumed to be 0.78, 0.65 and 0.97 respectively (Ernst et al., 1993, Zielman et al., 2017). In the calculation of the water attenuation factors for the occipital VOI the following T_1_ relaxation times of water; grey matter = 2130 ms, white matter = 1220 ms, CSF = 4425 ms and T_2_ relaxation times of water; grey matter = 50 ms, white matter = 55 ms, CSF = 141 ms, were used (Bartha et al., 2002, Marjańska et al., 2012, Rooney et al., 2007). The water attenuation was calculated separately for every subject based on the segmentation results of the corresponding VOI. Partial saturation due to T_1_ relaxation of the metabolites was not taken into account due to acquisition with a long TR. The T_2_ values of glutamate (93 ms) and other metabolites were taken from the literature (Marjańska et al., 2012).

### 2.7 Statistical analysis

Since no previous studies have explored changes in repeated glutamate assays before and during the initial phases of a provoked migraine-like attack, we could not rigorously estimate sample sizes. Therefore, we estimated that 15–20 participants with migraine were required, based on other studies that have reported measures of occipital Glx following different types of stimulation in migraine with study groups between 10 and 13 participants (Bridge et al., 2015, Siniatchkin et al., 2012). Previous GTN migraine-provocation studies performed by our group and others showed migraine-like attack incidence of around 80% for migraine without aura patients, therefore a required sample size of 25 migraineurs was determined (Ashina et al., 2017).

Values are presented as mean ± standard deviation (SD) for continuous data and numbers and percentages for categorical data. Normality and equality of variances were assessed with the Kolmogorov-Smirnov test and Levene’s test, respectively. Differences between the study groups in clinical characteristics, demographic characteristics and glucose concentrations per time point were tested using a Chi-square test for proportions, a Mann-Whitney test for non-normal distributed continuous variables, and an independent Student’s-test for normally distributed variables.

In the present study participants were scanned on baseline and 90 and 270 min after the start of GTN infusion (Fig. 1), as the onset time of a migraine-like attack is subject-dependent and cannot be exactly timed. Metabolite levels measured using ^1^H-MRS can be affected by factors such as diagnosis, migraine phase, timing of the scan session, age, baseline metabolite level, and response to GTN. In order to control for such factors and isolate the change in metabolite levels related to the transition from interictal into the preictal and ictal phase, we used a linear mixed model with the identity link function per metabolite (glutamate, glutamine, GABA, glutathione, myo-inositol, phosphoethanolamine, total creatine, total choline, total N-acetylaspartate, and aspartate). Metabolite concentration was the dependent variable; diagnosis (healthy controls/participants with migraine), scan session ([Baseline], [GTN-90], and [GTN-270]), migraine phase (determined for each participant on each individual scan session: interictal [prior to GTN infusion in GTN responders and also used for all scans from GTN nonresponders and for all healthy control scans in the model], preictal [defined as the period between GTN infusion and start of migraine-like headache onset if within 12 h after GTN infusion, independent of possible experienced nonheadache (premonitory) symptoms], ictal [migraine-like headache], and postictal as illustrated in Fig. 1D), and scan session-diagnosis interaction (to allow for different responses to GTN between healthy controls and migraineurs) were fixed factors. Age and baseline metabolite level were included in the model as a covariates. We used random effects for phase within subject with an unstructured correlation. Metabolite concentrations across the study were represented by the calculated estimated marginal mean at each scan session with 95% confidence intervals for each participant group. The outcomes were not controlled for multiple comparisons, and p-values < 0.05 were considered significant. Statistical analysis were performed using SPSS (version 23.0, IBM SPSS Statistics for Windows, Armonk, NY: IBM Corp).

## 3 Results

### 3.1 Data assessment

**Fig. 2:**
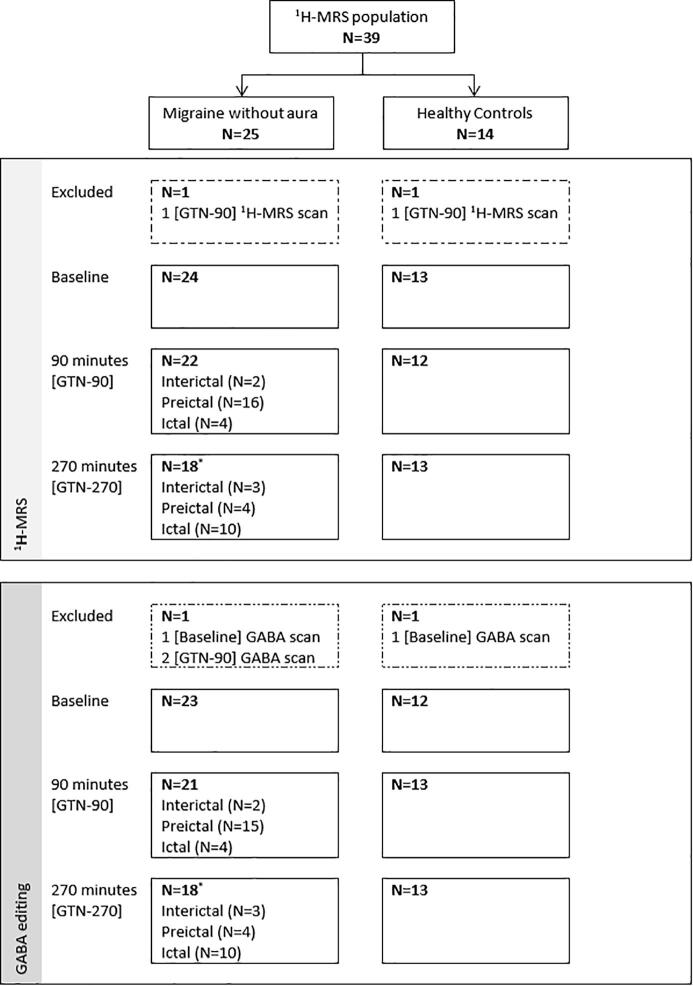
Flowchart for ^1^H-MRS and GABA editing analysis. Baseline = baseline scan session, GTN-90 = scan session 90 min after start glyceryl trinitrate infusion, GTN-270 = scan session 270 min after start glyceryl trinitrate infusion. Two participants were excluded (^1^H-MRS and GABA-edited) because no glyceryl trinitrate infusion was given (one participant with migraine dropped out due to claustrophobia after the 1st scan session and one healthy control dropped out due to logistic problems). * Included one patient in a postictal phase.

^1^H-MRS and GABA editing scans were obtained from 24 participants with migraine without aura and 13 healthy controls. Examples of pre and post GTN ^1^H-MRS spectra and GABA-edited spectra are shown in Fig. 1C. Two planned participants (one migraine without aura and one healthy control) who did not receive GTN infusion were excluded (Fig. 2). In spectral assessment, one ^1^H-MRS spectrum (healthy control [GTN-90]) and three GABA-edited spectra (one healthy control [Baseline] and two participants with migraine [Baseline] and [GTN-90]), were excluded from the analysis due to insufficient spectral quality. Two further spectra (^1^H-MRS and GABA-edited) from a participant with migraine [GTN-90] were excluded because the VOI was judged to be placed too far caudally, near the cerebellum. The reduction in acquired scans throughout the day is due to the development of migraine-like attacks; nausea and vomiting meant that some participants were unable to endure the entire procedure (Fig. 2).

The spectra included in the analysis had an average SNR of 58.22 ± 10.11 and a FWHM of the NAA peak of 0.039 ± 0.007 ppm, corresponding to 12 ± 2 Hz at baseline (measures reported by LCModel). The average creatine SNR of GABA-editing spectra measured 222.2 ± 48.5 at baseline. Note that the first reported SNR defined by LCModel is different from the SNR definition in the custom-written Matlab script for GABA fitting; therefore thresholds were adapted to the average reported SNR of each analysis software (LCModel or custom Matlab script). For all quality measures within the three scan sessions, see e-table 3, supplementary materials.

### 3.2 Clinical characteristics

**Fig. 3:**
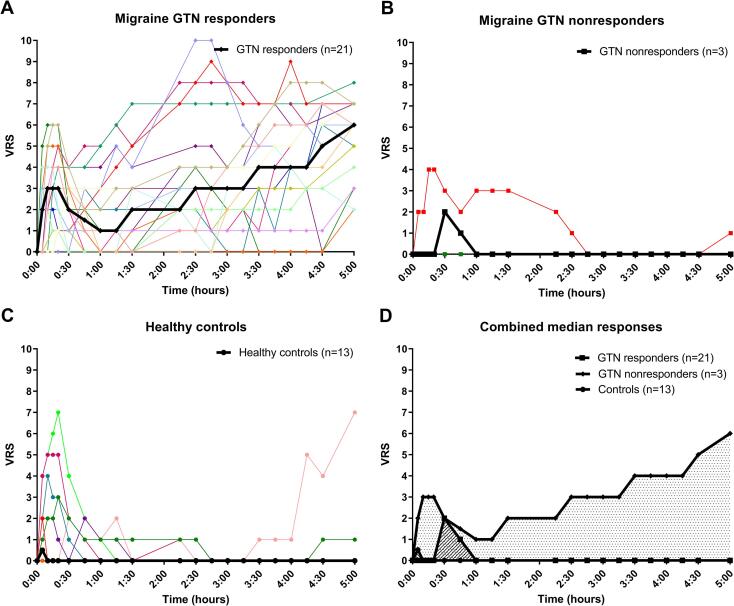
Verbal rating scale over time. GTN = glyceryl trinitrate, VRS = verbal rating scale. (A) Individual and median VRS are depicted for participants with migraine who developed a migraine-like attack after glyceryl trinitrate (GTN responders; individual cases = coloured lines with diamonds, median = black line with diamonds). (B) Individual and median VRS are depicted for participants with migraine who did not develop a migraine-like attack after glyceryl trinitrate (GTN nonresponders; individual cases = coloured lines with squares, median = black line with squares). (C) Individual and median VRS are depicted for healthy controls after glyceryl trinitrate infusion (individual cases = coloured lines with circles, median = black line with circles). (D) Median VRS are depicted for; GTN responders (dot fill-pattern), GTN nonresponders (right-diagonal line fill-pattern), and healthy controls (left-diagonal fill-pattern). Note, one control reported a high VRS at the end of the study. Clinically there was a discrepancy between the this VRS and how this affected the participant. Therefore, we do not regard this as a migraine-like headache. To validate our findings we performed a sensitivity analysis excluding this participant which had only marginal effects on the magnetic resonance spectroscopy outcomes and did not affect our main findings (e-table 7, supplementary materials).

Clinical characteristics and demographics from the study participants are shown in Table 1. Among these variables, there were no differences between groups except for the average headache days per month, which was higher for migraineurs. Ninety minutes after the start of GTN infusion mean systolic and diastolic blood pressure in participants with migraine (108.7 ± 15.4/71.5 ± 9.9 mmHg) and healthy controls (107.6 ± 10.2/69.3 ± 6.1 mmHg) declined compared with blood pressure before GTN infusion. No differences in blood pressure and heart rate were detected across the study day between study groups (e-table 4, supplementary materials). Blood glucose levels measured directly after each scan session revealed no differences between participants with migraine and healthy controls. There were no statistically significant differences in grey matter, white matter, or CSF content in the VOI between participants with migraine without aura and healthy controls (e-table 4, supplementary materials). Data on headache severity experienced by participants during and following GTN infusion is provided in Fig. 3. Seven out of 13 healthy controls (53.8%) experienced headache, but none of the 13 healthy controls developed a migraine-like attack. Twenty-one out of 24 participants with migraine (87.5%) experienced migraine-like attacks following GTN infusion and were defined as GTN responders. The three remaining participants with migraine that did not develop migraine-like attacks were defined as GTN nonresponders. Migraine-like attack characteristics following GTN infusion are shown in Table 2. In 20 out of 21 responders (95.2%) the migraine-like attack mimicked their usual migraine attacks. The median time of onset for migraine-like attacks was 190 min (range 45 – 345 min). One participant with migraine reported short term visual complaints (<10 min) during GTN infusion that did not resemble a migraine aura. No further visual, sensory, aphasic or motor symptoms were expressed by participants. In total 19 out of 21 GTN responders reported nonheadache (premonitory) symptoms prior to attack onset (e-Fig. 1, supplementary materials). None of the participants who developed a migraine-like attack took acute migraine medication prior to completing their final MRS scan session.

**Table 1:** Baseline characteristics of the study population receiving GTN.

Participants Characteristics	Migraine without aura	Healthy controls
	(n = 24)	(n = 13)
**General characteristics**		
Female	24 (100%)	13 (100%)
Age	36.2 ± 8.1	31.0 ± 9.0
BMI	23.7 ± 2.5	22.7 ± 1.8
Smoking (n, %)	2 (8.3%)	1 (7.7%)
**Migraine characteristics**		
Age of onset	16.1 ± 6.2	
Attack frequency (attack/month)	2.7 ± 1.1	
Attack duration treated (hours)	18.9 ± 25.7	
Headache days (days/month)	6.9 ± 3.5	0.5 ± 0.5
**Physiological measurements**		
Systolic blood pressure (mmHg)	120.2 ± 15.2	124.8 ± 10.8
Diastolic blood pressure (mmHg)	81.0 ± 11.1	78.8 ± 9.5
Heart rate (beats/min)	66.7 ± 8.2	65.5 ± 9.5
Baseline glucose (mmol/l)	4.7 ± 0.5	4.9 ± 0.7
**Tissue segmentation of VOI**		
GM fraction	0.59 ± 0.03	0.60 ± 0.04
WM fraction	0.35 ± 0.04	0.35 ± 0.04
CSF fraction	0.06 ± 0.03	0.05 ± 0.04

BMI = Body mass index, CSF = Cerebrospinal fluid, GM = Grey matter, GTN = Glyceryl trinitrate, VOI = volume of interest, WM = White matter. Values are expressed as absolute values and percentage or mean ± SD.

**Table 2:** Migraine-like attack characteristics in GTN responders following GTN infusion.

ID	Headache characteristics^a^	Associated symptoms^b^	Mimics usual migraine	Migraine-like attack onset	Peak headache /pain score (time)^c^	Treatment [time]/efficacy
P2	6/Right/Stab/+	+/-/+/-	Yes	210 min	8 (240 min)	Paracetamol 1 g with caffeine (100 mg) [at 15:45 h], and again at 18:00 h/yes
P3	5/Left/Throb/+	+/-/-/-	Yes	195 min	7 (270 min)	Zolmitriptan 5 mg [at 15:20 h]/yes
P4	4/Bilat/Throb/+	-/-/+/+	Yes	210 min	6 (300 min)	Not reported
P5	5/Right/Pres/+	-/-/+/-	Yes	225 min	7 (300 min)	Excedrin® (USA) [at 16:00 h], and [at 19:00 h]/NR
P7	4/Bilat/Pres/+	-/-/+/+	Yes	330 min	>4 (330 min)	No medication, went to sleep/yes
P8	5/Left/Throb/+	+/-/+/-	Yes	225 min	5 (225 min)	No medication
P9	3/Bilat/Pres/+	+/-/+/-	Yes	135 min	6 (225 min)	Relpax® 40 mg [at 16:00 h], Paracetamol 1 g 20:00 h/no
P10	3/Bilat/Pres/-	-/-/-/-	Yes	60 min	10 (150 min)	Imigran® injection [at 12:45 h], again [at 15:10 h]/yes
P12	3/Right/Pres/+	+/-/+/-	Yes	300 min	3 (300 min)	Sumatriptan [at 16:30 h]/yes
P13	5/Left/Throb/+	-/-/-/+	Yes	90 min	9 (300 min)	Sumatriptan 50 mg [at 12:50 h], and [at 15:22 h] Imigran® injection after vomiting/yes
P14	S/Bilat/Throb/+	+/-/+/+	No	345 min	S (345 min)	Paracetamol 1 g and naproxen 250 mg [at 16:30 h], Imigran® [at 17:15 h]/yes
P16	2/Left/Throb/+	-/-/+/-	Yes	195 min	5 (300 min)	Almogran® 12.5 mg [at ± 15:30 h] and went to sleep/yes
P17	3/Bilat/Pres/-	-/-/-/-	Yes	300 min	3 (300 min)	Sumatriptan 50 mg [at 15:10 h] and went to sleep/yes
P18	4/Right/Throb/+	+/+/-/-	Yes	45 min	8 (300 min)	Imigran® injection [at 11:13 h]/no
P19	6/Left/Stab/+	-/-/+/+	Yes	270 min	6 (270 min)	Alka-Seltzer® 3 tablets [at 15:30 h], and again [at 22:00 h]/no
P20	3/Right/Throb/-	-/-/-/-	Yes	150 min	7 (300 min)	Sumatriptan 100 mg [at 15:00 h], Imigran® injection [at 16:15 h]/yes
P21	4/Bilat/Pres/-	-/-/+/-	Yes	135 min	7 (285 min)	Sumatriptan injection [at 15:00 h], Sumatriptan injection [at 18:30 h], Arcoxia® [at 22:00 h]/yes
P22	4/Left/Throb/-	+/-/-/-	Yes	45 min	5 (150 min)	No medication
P23	5/Bilat/Pres/+	-/-/+/-	Yes	45 min	8 (135 min)	Imigran® injection [at 13:15 h], Advil® 200 mg [at 23:00 h]/no
P24	4/Bilat/Throb/-	-/-/-/-	Yes	240 min	6 (300 min)	Relpax® 40 mg [at 15:15 h], APC 1 g [at 18:00 h]/yes
P25	5/Right/Throb/-	-/-/-/-	Yes	240 min	7 (300 min)	Rizatriptan 10 mg [at 15:30 h]/yes

a Verbal pain score/localization/pain quality/aggravation by movement. In case no migraine-like headache was provoked during the study day, headache characteristics on the last standard questionnaire (on 300 min after GTN infusion) were listed.

b Nausea/vomiting/photophobia/phonophobia.

c In case the migraine-like attack was still developing the peak headache intensity at 300 min was provided. APC = acetylsalicylic acid, paracetamol and caffeine, Bilat = bilateral, NR = not reported, Pres = pressing, S = severe, Stab = stabbing, Throb = throbbing/pounding.

### 3.3 Magnetic resonance spectroscopy

**Fig. 4:**
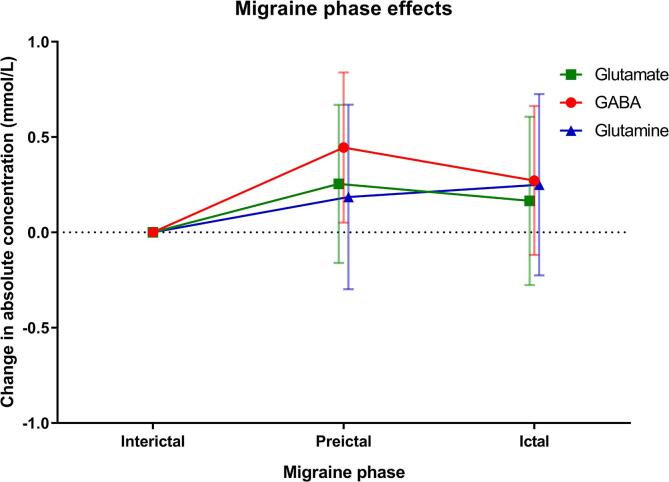
^1^H-MRS metabolite changes across the migraine phases taking control-data into account. Results from the linear mixed-effect model for repeated measures with metabolite concentration as dependent variable, fixed factors (diagnosis (healthy controls/migraineurs), scan session ([Baseline], [GTN-90], and [GTN-270]), migraine phase (interictal, preictal, ictal, postictal), and scan session-diagnosis interaction) and age as covariate. Fixed effects estimates for the change in glutamate (green), GABA (red), and glutamine (blue) levels from baseline to preictal and ictal phase are plotted. The measurements of the controls and nonresponders were always classified as “interictal”. Baseline = baseline scan session. Error bars show 95% CI. The transition from the interictal phase to either the preictal or ictal phase of GTN provoked migraine-like attacks had no significant influence on glutamate (p = 0.222 and p = 0.454) and glutamine (p = 0.441 and p = 0.293), but revealed that the transition from the interictal to preictal phase led to increase in GABA level (p = 0.028).

First, we determined average metabolite concentrations per scan session for responders, nonresponders and healthy controls (e-table 5, supplementary materials). This approach is limited to being able to detect metabolite changes specifically related to migraine-like attack onset when using fixed scan sessions (Baseline, GTN-90, and GTN-270) because the time to migraine-like attack onset varied between responders (Table 2 and e-Fig. 2, supplementary materials). In order to identify metabolites involved in the onset of a migraine-like attack, we used a mixed model approach. In the model we corrected for age, diagnosis (migraine case – healthy control), scan session (Baseline – GTN-90 – GTN-270), and scan session-diagnosis interaction (to allow for different responses to GTN between study groups) to isolate metabolite changes specifically related to migraine-like attack onset, see e-table 6, supplementary materials. The transition from the interictal phase to either the preictal or ictal phase of GTN provoked migraine-like attacks had no influence on glutamate concentrations (p = 0.222 and p = 0.454) or glutamine concentrations (p = 0.441 and p = 0.293; Table 3 and Fig. 4). Analysis of the GABA concentrations showed that the transition from the interictal to preictal phase led to an increase in GABA level (p = 0.028; Table 3 and Fig. 4). Sensitivity analysis by excluding either; the one GTN responder with a postictal phase (Fig. 2), two responders without nonheadache (premonitory) symptoms (e-Fig. 1), or a healthy control with a high VRS at the end of the study day (Fig. 3) did not affect this finding.

**Table 3:** Migraine phase effects.

Metabolite	Migraine phase effects			
	Change from baseline to preictal phase		Change from baseline to ictal phase	
	Estimate (95% CI)	p-value	Estimate (95% CI)	p-value
Glutamate	0.25 (-0.16 – 0.67)	0.222	0.17 (-0.28 – 0.61)	0.454
Glutamine	0.19 (-0.30 – 0.67)	0.441	0.25 (-0.23 – 0.73)	0.293
GABA	0.45 (0.05 – 0.84)	**0.028**	0.27 (-0.12 – 0.66)	0.167
GSH	−0.11 (-0.28 – 0.06)	0.215	−0.04 (-0.21 – 0.13)	0.621
tNAA	0.19 (-0.30 – 0.67)	0.439	0.17 (-0.31 – 0.65)	0.473
tCr	0.06 (-0.26 – 0.38)	0.728	0.10 (-0.21 – 0.42)	0.505
Ins	−0.02 (-0.35 – 0.31)	0.891	0.05 (-0.28 – 0.38)	0.763
tCho	0.03 (-0.04 – 0.09)	0.386	0.004 (-0.06 – 0.07)	0.882
Aspartate	0.11 (-0.48– 0.70)	0.709	−0.23 (-0.80 – 0.33)	0.410
PE	0.23 (-0.03 – 0.49)	0.084	0.17 (-0.06 – 0.41)	0.145

GSH = glutathione, Ins = myo-inositol, PE = phosphoethanolamine, tCr = total creatine, tCho = total choline, tNAA = total *N*-acetylaspartate. Values are expressed as mean mmol/L and 95% confidence intervals. p-values < 0.05 in bold.

## 4 Discussion

We hypothesized involvement of the glutamatergic system in the initiation of migraine attacks either by a solitary elevation of glutamate levels or via a broader involvement through an excitatory-inhibitory disbalance with GABA. We have used 7 Tesla ^1^H-MRS to measure glutamate, glutamine and GABA levels in the visual cortex over the course of GTN-provoked attacks in female migraineurs and in healthy controls. We did not observe a change in glutamate and glutamine levels when migraineurs transited from interictal to the preictal and ictal state, however, we did observe increased GABA levels in the preictal phase in migraineurs compared with healthy controls. This observation suggests that the increase in GABA concentration is associated with the onset of a migraine attack.

It seems unlikely that the observed interictal to preictal increase in GABA is due to diurnal influences, as GABA levels were shown to be stable during the day (Evans et al., 2010). Although previous MRS studies on interictal GABA measurements have presented conflicting results, a recent *meta*-analysis suggested an increased GABA level in interictal migraineurs, while in musculoskeletal pain and other chronic pain syndromes no elevation was found (Peek et al., 2020). During the ictal phase GABA also appears to be elevated in CSF (van Dongen et al., 2016). To our knowledge no ictal GABA measurements have been performed using MRS. Our findings therefore strongly suggest an evident role of GABA in the migraine pathophysiology. We speculate that increased preictal GABA levels, as observed in our study, may reflect a compensating mechanism to reduce an hyperexcitatory state and/or may reflect a protective role for GABA in suppressing headaches (Bigal et al., 2008, Watson, 2016).

Only a few ^1^H-MRS studies have investigated metabolite concentrations prior to or during the migraine attack, (Arngrim et al., 2016, Jacob et al., 2006, Younis et al., 2020) but these studies did not report separately on glutamine and glutamate, and did not assess GABA levels (Arngrim et al., 2016, Younis et al., 2020). Two studies measured Glx, in the visual cortex (migraine with aura) and pons (migraine without aura) during provoked migraine-like attacks through hypoxia, calcitonin gene-related peptide (CGRP) and sildenafil, and revealed no change in Glx levels (Arngrim et al., 2016, Younis et al., 2020). This is in line with our observation that glutamate and glutamine levels did not change in the preictal or ictal state of provoked migraine-like attacks.

There are several possible physiological explanations why changes in glutamate levels were not detected during attack initiation. As only female migraine without aura patients were included, we formally still not exclude glutamate level changes in migraine with aura attacks, although GTN provocation studies have only rarely been able to provoke aura symptoms, even in migraine patients with (hemiplegic) aura, and it is known that GTN does not provoke migraine-like headache in hemiplegic migraine patients (Ashina et al., 2017). This suggests that the effect of GTN is further down the pathophysiological cascade of events, leading only to the onset of migraine-like headache but not migraine aura, which is caused by CSD (Ferrari et al., 2015). The following pathway to provoke migraine-like headache has currently been proposed for GTN; nitric oxide released by GTN activates intracellular soluble guanylate cyclase, which catalyzes the formation of cyclic guanosine monophosphate (cGMP) an important second messenger involved in the activation of various protein kinases, implicated in smooth muscle relaxation and vasodilatation (Ashina et al., 2017). In CSD induction and/or propagation glutamate is suspected to bind to the N-methyl-D-aspartate receptor (NMDA) receptor which may cause an increase in intracellular calcium, which in turn binds to calmodulin and activates neuronal nitric oxide synthase (NOS), which is also able to produce and increase nitric oxide levels (Pietrobon and Moskowitz, 2014, Pradhan et al., 2018). That rise in nitric oxide level, may activate aforementioned intracellular soluble guanylate cyclase, resulting in cGMP formation thought to be involved in migraine-like headache. Taken together, this may suggest that infusion with GTN, a nitric oxide donor, bypasses the glutamate–nitric oxide–cGMP pathway as it directly engages nitric oxide.

In a 3 Tesla study of healthy controls a transient rise in brainstem Glx levels was found after administration of sildenafil, independent of provoked headache (Younis et al., 2018). Sildenafil is a selective inhibitor of the phosphodiesterase 5 enzyme, which breaks down cGMP, and is expected to cause cGMP accumulation (Ashina et al., 2017). While CGRP, that triggers migraine via the cyclic adenosine monophosphate pathway, did not induce Glx changes in the brainstem or thalamus (Younis et al., 2018). Our and previous findings (Younis et al., 2018) show the importance of including healthy controls in provocation studies in order to ensure that direct pharmacological effects of the provocation substance itself is not incorrectly labeled as a marker for provoked attacks. It also shows that studying spontaneous attacks instead of provoked attacks has the advantage of not including pharmacological effects of the provocative substance, as well as a lower risk of bypassing part of the attack-initiation pathophysiological pathway. Another option might be to select other provocation models, for instance those which act on ion channels (Al-Karagholi et al., 2019).

Previously we found an elevated interictal glutamate concentration in the visual cortex in migraineurs (Zielman et al., 2017). In the current study we did not statistically test for this because it was not an objective of this study and we concluded that the study was underpowered to replicate the previous finding. However, for our main objective, with an intra-individual follow-up study, our power was adequate. Furthermore, in the current study we included only females, while the previous study included both males and females. It is further good to note when comparing different studies that the reported absolute concentrations are influenced by metabolite fitting, baseline smoothness and quantification methods (Bhogal et al., 2017, Marsman et al., 2017, Mosconi et al., 2014, Zhang and Shen, 2014).

Our study is not without limitations. Firstly, some migraineurs were unable to be scanned during the headache phase due to nausea and vomiting, a general problem when studying migraine attacks. This may have introduced a selection bias towards attenuation of metabolite changes related to the headache phase of the migraine attack. Furthermore, a potential additional source of selection bias may be that participants that experience phonophobia during their migraine attack might less willing to participate in the study due to MR noise, although none of the migraine patients that we approached refrained from participation because of this. Secondly, the primary investigator who acquired the scans was not blinded for participant status, which may have introduced bias in placement of the VOI. However, clear anatomical landmarks were used, and placement was checked by an independent (blinded) observer. Spectral assessments were scored blindly. Thirdly, we did not include a placebo group either as a separate group or in a crossover design, however, the downside is that GTN typically gives rise to immediate (infusion) headache also seen in our control group which risks unblinding the participants. Furthermore, in a crossover design this would entail submitting participants to another intensive and burdensome study day. Fourthly, we included only females which may limit generalizability of our findings. Fifthly, despite that we acquired large number of MRS scans in a repeated measures fashion enabling metabolite concentrations to be measured during attack development, we cannot fully exclude the possibility that the study might be underpowered to detect subtle differences. However, the 95% confidence intervals indicate the changes in interictal glutamate levels between the preictal or ictal state probably lie roughly between −0.30 – 0.70 mmol/L implying between a −3.2% to a + 7.7% change in glutamate. In a previous study we detected a 9.7–10.5% (0.62–0.67 mmol/L) elevation in glutamate levels comparing interictal migraine without aura with healthy controls (Zielman et al., 2017). Therefore, we feel confident that our study was sufficiently well-powered to study the different phases of provoked attacks. Sixthly, because we positioned the VOI in the visual cortex, our findings cannot be extrapolated to other brain regions. Lastly, isolated small extracellular changes in glutamate cannot be measured with this MRS technique as synaptic glutamate levels are very low when compared to the over 10000-fold higher intracellular levels (Erecinska and Silver, 1990). Therefore, for instance, subtle local synaptic changes in glutamate levels cannot be ruled out, nor can shifts between de different glutamate pools. Other techniques such as dynamic carbon (^13^C-MRS) with infusion of ^13^C-enriched glutamate substrates, which enable tracking novel metabolite formation, might be useful to assess fluxes in the glutamate-glutamine/GABA cycle (Walls et al., 2014).

In conclusion, we have evaluated the glutamatergic system with 7 Tesla single-volume ^1^H-MRS in the visual cortex in the evolution from interictal status towards the initial phases of provoked migraine-like attacks in migraine patients without aura and compared these results with matched healthy controls. Glutamate and glutamine levels showed no change from interictal to the preictal and ictal state, but GABA levels increased from interictal to the preictal state in migraineurs. We conclude that high resolution 7 T MRS is able to show changes in the glutamatergic system in response to a triggered migraine attack, revealing an increase in GABA concentration associated with the onset of a migraine attack. This association may support the hypothesis that susceptibility to develop migraine attacks is related to dysregulation of excitability through an excitatory-inhibitory disbalance with GABA.

### CRediT authorship contribution statement

**Gerrit L.J. Onderwater:** Conceptualization, Methodology, Formal analysis, Investigation, Data curation, Writing – original draft, Writing – review & editing, Visualization. **Jannie P. Wijnen:** Conceptualization, Methodology, Software, Resources, Data curation, Writing – review & editing. **Chloé Najac:** Software, Formal analysis, Data curation, Writing – review & editing. **Robin M. van Dongen:** Data curation, Writing – review & editing. **Itamar Ronen:** Software, Resources, Data curation, Writing – review & editing, Supervision. **Andrew Webb:** Resources, Writing – review & editing, Supervision. **Ronald Zielman:** Conceptualization, Writing – review & editing. **Erik W. van Zwet:** Formal analysis, Writing – review & editing, Supervision. **Michel D. Ferrari:** Conceptualization, Resources, Writing – review & editing, Supervision, Funding acquisition. **Hermien E. Kan:** Conceptualization, Methodology, Writing – review & editing, Supervision. **Mark C. Kruit:** Conceptualization, Methodology, Writing – review & editing, Supervision. **Gisela M. Terwindt:** Conceptualization, Methodology, Writing – review & editing, Supervision, Funding acquisition.

## Declaration of Competing Interest

A.G. Webb reports independent support from Dutch Research Council (NWO) and the European Research Council. M.D. Ferrari reports grants and consultancy or industry support from Medtronic, Novartis, Amgen, Lilly, Teva, electroCore and independent support from Dutch Research Council , NIH, European Community, and the Dutch Heart Foundation. H.E. Kan reports independent grants from NWO, Duchenne Parent Project, and the EU, consultancy for PTC therapeutics and trial support from ImagingDMD outside the submitted work. R. Zielman reports support for conference visits from Menarini and Allergan, and is currently an employee at Novartis. G.M. Terwindt reports grants or consultancy support from Novartis, Lilly, Teva, Allergan, and independent support from the Dutch Research Council (ZonMW) the Dutch Heart Foundation, and Dutch Brain Foundation. The other authors report no conflicts of interest.
