# Supplementary material for: Cortical glutamate and gamma-aminobutyric acid over the course of a provoked migraine attack, a 7 Tesla magnetic resonance spectroscopy study
Source: Neuroimage Clin. 2021 Nov 24;32:102889. doi: 10.1016/j.nicl.2021.102889 (PMC8640106; doi:10.1016/j.nicl.2021.102889)
Supplement: Supplementary data 1 [file mmc1.docx]

**Supplementary figures and tables**

E-Figure 1: Reported nonheadache symptoms over time (0-5h) by individual GTN responders

E-Figure 2: Timing migraine onset in GTN responders.

E-Table 1: Applied LCModel control parameters

E-Table 2: Migraine phase effects with the full basis set

E-Table 3: General quality measures and CRLBs from included metabolites

E-Table 4: Physiological measurements and VOI characteristics during the study day for included 1H-MRS scans

E-Table 5: Average metabolite concentrations over time independent of migraine phase

E-Table 6: Linear mixed-effect model correction factors

E-Table 7: Migraine phase effects with exclusion of one control subject with high verbal rating scale

**
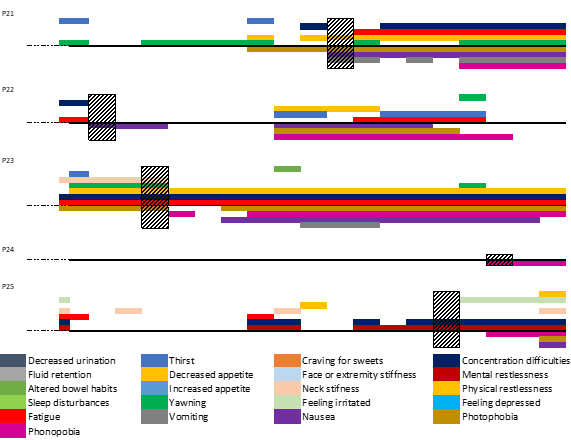
**

**E-Figure 1:** Reported nonheadache symptoms over time (0-5h) by individual GTN responders, each participant is represented by single line (interrupted line: during GTN infusion, continuous line: post-GTN infusion) with each nonheadache symptom represented by a different color (see legend). Nonheadache symptoms part of the associated criteria for migraine are represented below the line, other symptoms above. When a particular nonheadache symptom was reported directly prior and directly after MR scanning (no questionnaire) the symptom considered to be present during scanning in this representation. The onset for migraine-like headache is marked by a diagonally marked interval.

**E-Figure 2:** Timing migraine onset in GTN responders. The onset of migraine is plotted for each glyceryl trinitrate (GTN) responder with respect to the fixed scan sessions 90 and 270 minutes after GTN infusion. Note that not each patient was indeed scanned at each scan session, as scans were cancelled due to nausea and vomiting in patients. For the numbers of GTN responders scanned and included in the analysis see Figure 2 study flowchart.

**E-Table 1: Applied LCModel control parameters**

| **LCModel control parameter** | **Value** |
| --- | --- |
| atth2o | 1 |
| attmet | 1 |
| chomit(1) | 'Ala' |
| chomit(2) | 'Asc' |
| chomit(3) | 'GABA' |
| chomit(4) | 'Gly' |
| chomit(5) | 'Lac' |
| chomit(6) | 'PCho' |
| chomit(7) | 'Ser' |
| chomit(8) | 'Thr' |
| chomit(9) | 'MM09' |
| chomit(10) | 'Lip13a' |
| chomit(11) | 'H20' |
| deltat | 2.500e-04 |
| dkntmn | 1 |
| dows | T |
| echot | 36.00 |
| hzpppm | 2.9806e+02 |
| ncombi | 18 |
| neach | 50 |
| nomit | 11 |
| nsimul | 1 |
| nunfil | 2048 |
| ppmend | 0.2 |
| ppmst | 4.2 |
| wconc | 45556 |
|  | |

**E-Table 2: Migraine phase effects with the full basis set**

| **Metabolite** | **Migraine phase effects** | | | |
| --- | --- | --- | --- | --- |
|  | Change from baseline to preictal phase |  | Change from baseline to ictal phase |  |
|  | Estimate (95% CI) | p-value | Estimate (95% CI) | p-value |
| Glutamate | 0.30 (-0.13 – 0.72) | 0.169 | 0.19 (-0.27 – 0.65) | 0.413 |
| Glutamine | 0.14 (-0.43 – 0.71) | 0.619 | 0.28 (-0.28 – 0.85) | 0.314 |
| GABA | 0.45 (0.05 – 0.84) | **0.028** | 0.27 (-0.12 – 0.66) | 0.167 |
| GSH | -0.10 (-0.33 – 0.14) | 0.410 | -0.02 (-0.26 – 0.22) | 0.883 |
| tNAA | 0.21 (-0.25 – 0.68) | 0.352 | 0.21 (-0.24 – 0.67) | 0.350 |
| tCr | 0.03 (-0.29 – 0.35) | 0.833 | 0.07 (-0.25 – 0.38) | 0.667 |
| Ins | 0.09 (-0.38 – 0.56) | 0.691 | 0.13 (-0.32 – 0.58) | 0.565 |
| tCho | 0.07 (-0.06 – 0.20) | 0.282 | 0.07 (-0.05 – 0.20) | 0.258 |
| Aspartate | 0.03 (-0.65– 0.70) | 0.934 | -0.45 (-1.14 – 0.24) | 0.197 |
| PE | 0.38 (-0.62 – 1.38) | 0.444 | -0.12 (-1.13 – 0.89) | 0.806 |
| Values are expressed as mean mmol/L and 95% confidence intervals. p-values < 0.05 in bold. GSH = glutathione, Ins = myo-inositol, PE=phosphoethanolamine, tCr= total creatine, tCho = total choline, tNAA = total *N*-acetylaspartate. | | | | |

**E-Table 3: General quality measures and CRLBs from included metabolites**

| **Quality measures** | **Baseline** | **90 min**  **[GTN-90]** | **270 min**  **[GTN-270]** |
| --- | --- | --- | --- |
| **^1^H-MRS quality measures** |  |  |  |
| SNR | 58.22 ± 10.11 | 59.47 ± 5.60 | 59.35 ± 7.30 |
| Full-width at half maximum NAA (Ppm) | 0.039 ± 0.007 | 0.040 ± 0.006 | 0.038 ± 0.007 |
| **^1^H-MRS metabolites** |  |  |  |
| CRLB Glutamate | 2.14 ± 0.54 | 2.03 ± 0.17 | 2.06 ± 0.25 |
| CRLB Glutamine | 11.43 ± 4.56 | 10.41 ± 2.39 | 10.26 ± 2.18 |
| CRLB GSH | 7.22 ± 2.42 | 6.76 ± 1.58 | 6.84 ± 1.16 |
| CRLB tNAA | 1.00 ± 0.00 | 1.00 ± 0.00 | 1.00 ± 0.00 |
| CRLB tCr | 1.03 ± 0.16 | 1.00 ± 0.00 | 1.00 ± 0.00 |
| CRLB Ins | 2.08 ± 0.49 | 2.00 ± 0.00 | 2.00 ± 0.00 |
| CRLB tCho | 3.30 ± 0.46 | 3.35 ± 0.65 | 3.29 ± 0.46 |
| CRLB PE^a^ | 10.44 ± 3.16 | 5.32 ± 0.68 | 5.55 ± 0.81 |
| CRLB Aspartate | 12.62 ± 13.61 | 10.00 ± 3.37 | 10.03 ± 3.37 |
| **GABA-edited ^1^H-MRS quality measures** |  |  |  |
| Creatine SNR | 222.24 ± 48.50 | 219.07 ± 48.51 | 220.54 ± 42.95 |
| **GABA-edited ^1^H-MRS metabolites** |  |  |  |
| CRLB GABA | 3.44 ± 0.99 | 3.60 ± 0.96 | 3.31 ± 0.81 |
| ^a^ Excluding one scan with an undeterminable PE peak at baseline with this scan PE =32.43 ± 163.32. Values are expressed as mean ± SDs. Baseline = baseline scan session, CRLB = Cramér-Rao lower bound, GABA = gamma-aminobutyric acid, GSH = glutathione, GTN-90 = scan session 90 minutes after start glyceryl trinitrate infusion, GTN-270 = scan session 270 minutes after start glyceryl trinitrate infusion, Ins = myo-inositol, PE = phosphoethanolamine, tCho = total choline, tCr = total creatine, tNAA = total *N*-acetylaspartate, SNR = Signal to noise ratio. | | | |

**E-Table 4: Physiological measurements and VOI characteristics during the study day for included ^1^H-MRS scans**

| **Participants Characteristics** | **Baseline** | | | **90 minutes [GTN-90]** | | | **270 minutes [GTN-270]** | | |
| --- | --- | --- | --- | --- | --- | --- | --- | --- | --- |
|  | Migraine without aura | Healthy controls | p-value | Migraine without aura | Healthy controls | p-value | Migraine without aura | Healthy controls | p-value |
|  | (n=24) | (n=13) |  | (n=22) | (n=12) |  | (n=18) | (n=13) |  |
| **Physiological measurements** |  |  |  |  |  |  |  |  |  |
| Blood pressure (mmHg) |  |  |  |  |  |  |  |  |  |
| Systolic | 120.2 ± 15.2 | 124.8 ± 10.8 | 0.347^b^ | 108.7 ± 15.4 | 107.6 ± 10.2 | 0.827^b^ | 110.4 ± 15.0 | 110.9 ± 10.1 | 0.907^b^ |
| Diastolic | 81.0 ± 11.1 | 78.8 ± 9.5 | 0.551^b^ | 71.5 ± 9.9 | 69.3 ± 6.1 | 0.488^b^ | 74.5 ± 10.9 | 72.5 ± 8.2 | 0.588^b^ |
| Heart rate (beats/min) | 66.7 ± 8.2 | 65.5 ± 9.5 | 0.689^b^ | 67.6 ± 12.5 | 61.3 ± 9.3 | 0.170^b^ | 75.1 ± 8.5 | 68.5 ± 12.5 | 0.111^b^ |
| Glucose (mmol/l) | 4.7 ± 0.5 | 4.9 ± 0.7 | 0.534^a^ | 4.9 ± 0.8 | 4.6 ± 0.7 | 0.492^a^ | 5.2 ± 0.9 | 5.2 ± 1.2 | 0.236^a^ |
| **Tissue segmentation of VOI** |  |  |  |  |  |  |  |  |  |
| GM fraction | 0.59 ± 0.03 | 0.60 ± 0.04 | 0.616^b^ | 0.60 ± 0.04 | 0.62 ± 0.04 | 0.222^b^ | 0.61 ± 0.03 | 0.61 ± 0.04 | 0.958^b^ |
| WM fraction | 0.35 ± 0.04 | 0.35 ± 0.04 | 0.810^b^ | 0.35 ± 0.04 | 0.34 ± 0.05 | 0.459^b^ | 0.33 ± 0.04 | 0.34 ± 0.04 | 0.521^b^ |
| CSF fraction | 0.06 ± 0.03 | 0.05 ± 0.04 | 0.102^a^ | 0.05 ± 0.02 | 0.05 ± 0.04 | 0.118^a^ | 0.06 ± 0.03 | 0.05 ± 0.05 | 0.055^a^ |

Physiological measures at 90 minutes after glyceryl trinitrate infusion had 2 participants with migraine and 2 healthy controls missing heart information. Physiological measures at 270 minutes after glyceryl trinitrate infusion had 1 participant with migraine missing blood pressure, and 3 participants with migraine missing heart information. ^a^Mann-Whitney U test, ^b^Student’s T-test. Values are expressed as mean ± SD. p-values < 0.05 are in bold. Baseline = baseline scan session, CSF = Cerebrospinal fluid, GM = Grey matter, GTN = Glyceryl trinitrate, GTN-90 = scan session 90 minutes after start glyceryl trinitrate infusion, GTN-270 = scan session 270 minutes after start glyceryl trinitrate infusion, VOI = volume of interest, and WM = white matter.

**E-Table 5:** **Average metabolite concentrations over time independent of migraine phase**

| **Metabolite** | **Participants** | **Baseline** | **90 min**  **[GTN-90]** | **270 min**  **[GTN-270]** |
| --- | --- | --- | --- | --- |
| Glutamate | Healthy controls | 8.76 ± 0.56 | 8.72 ± 0.51 | 8.86 ± 0.66 |
|  | GTN responders | 8.81 ± 0.56 | 8.67 ± 0.50 | 8.81 ± 0.72 |
|  | GTN non-responders | 8.20 ± 0.14 | 7.95 ± 0.25 | 8.22 ± 0.06 |
| Glutamine | Healthy controls | 2.30 ± 0.40 | 2.43 ± 0.45 | 2.46 ± 0.43 |
|  | GTN responders | 2.30 ± 0.53 | 2.37 ± 0.36 | 2.44 ± 0.44 |
|  | GTN non-responders | 2.00 ± 0.23 | 2.19 ± 0.17 | 2.15 ± 0.34 |
| GABA | Healthy controls | 3.28 ± 0.67 | 3.50 ± 0.45 | 3.13 ± 0.49 |
|  | GTN responders | 3.34 ± 0.48 | 3.23 ± 0.40 | 3.29 ± 0.46 |
|  | GTN non-responders | 3.17 ± 0.28 | 2.90 ± 0.40 | 2.91 ± 0.23 |
| GSH | Healthy controls | 1.25 ± 0.15 | 1.25 ± 0.17 | 1.23 ± 0.12 |
|  | GTN responders | 1.13 ± 0.18 | 1.11 ± 0.18 | 1.13 ± 0.17 |
|  | GTN non-responders | 1.05 ± 0.13 | 1.11 ± 0.02 | 1.16 ± 0.24 |
| tNAA | Healthy controls | 11.59 ± 0.72 | 11.96 ± 0.64 | 11.77 ± 0.81 |
|  | GTN responders | 11.64 ± 0.58 | 11.63 ± 0.54 | 11.75 ± 0.63 |
|  | GTN non-responders | 11.80 ± 0.11 | 11.49 ± 0.35 | 11.63 ± 0.60 |
| tCr | Healthy controls | 7.36 ± 0.39 | 7.57 ± 0.35 | 7.45 ± 0.35 |
|  | GTN responders | 7.34 ± 0.40 | 7.39 ± 0.48 | 7.41 ± 0.43 |
|  | GTN non-responders | 7.07 ± 0.31 | 7.21 ± 0.58 | 7.10 ± 0.17 |
| Ins | Healthy controls | 4.99 ± 0.39 | 5.16 ± 0.48 | 5.03 ± 0.47 |
|  | GTN responders | 4.76 ± 0.52 | 4.82 ± 0.46 | 4.84 ± 0.36 |
|  | GTN non-responders | 4.60 ± 0.52 | 4.66 ± 0.45 | 4.69 ± 0.58 |
| tCho | Healthy controls | 0.68 ± 0.10 | 0.67 ± 0.13 | 0.67 ± 0.09 |
|  | GTN responders | 0.69 ± 0.09 | 0.67 ± 0.07 | 0.67 ± 0.07 |
|  | GTN non-responders | 0.60 ± 0.01 | 0.60 ± 0.05 | 0.60 ± 0.05 |
| PE | Healthy controls | 2.93 ± 0.19 | 3.18 ± 0.84 | 2.96 ± 0.12 |
|  | GTN responders | 2.75 ± 0.67 | 2.96 ± 0.26 | 2.98 ± 0.25 |
|  | GTN non-responders | 2.75 ± 0.15 | 2.91 ± 0.30 | 2.85 ± 0.07 |
| Aspartate | Healthy controls | 2.80 ± 0.59 | 3.14 ± 0.65 | 2.93 ± 0.42 |
|  | GTN responders | 2.58 ± 0.73 | 2.69 ± 0.57 | 2.76 ± 0.66 |
|  | GTN non-responders | 2.30 ± 0.50 | 2.43 ± 0.04 | 2.85 ± 0.72 |
| Values are absolute concentrations (uncorrected for migraine phase, age, and scan session-diagnosis interaction) per scan session, expressed as mean mmol/L ± SDs. Baseline = baseline scan session, GSH = glutathione, GTN-90 = scan session 90 minutes after start glyceryl trinitrate infusion, GTN-270 = scan session 270 minutes after start glyceryl trinitrate infusion, Ins = myo-inositol, PE = phosphoethanolamine, tCho = total choline, tCr = total creatine, tNAA = total *N*-acetylaspartate. | | | | |

**E-Table 6:** **Linear mixed-effect model correction factors**

| **Metabolite** | **Age** | **Diagnosis** | **Scan session** | **Diagnosis-scan session interaction** |
| --- | --- | --- | --- | --- |
|  | p-value | p-value | p-value | p-value |
| Glutamate | 0.437 | 0.673 | **0.004** | 0.596 |
| Glutamine | 0.236 | 0.424 | 0.875 | 0.680 |
| GABA | 0.221 | 0.203 | 0.126 | **0.010** |
| GSH | 0.658 | 0.174 | 0.696 | 0.713 |
| tNAA | 0.190 | 0.694 | 0.920 | 0.185 |
| tCr | **0.034** | 0.116 | 0.548 | 0.444 |
| Ins | 0.164 | 0.052 | 0.494 | 0.672 |
| tCho | **0.0003** | 0.143 | 0.540 | 0.509 |
| Aspartate | 0.628 | 0.391 | 0.212 | 0.132 |
| PE | 0.223 | 0.056 | 0.661 | 0.344 |

p-values < 0.05 in bold. GSH = glutathione, Ins = myo-inositol, PE=phosphoethanolamine, tCr= total creatine, tCho = total choline, tNAA = total *N*-acetylaspartate.

**E-Table 7: Migraine phase effects with exclusion of one control subject with high verbal rating scale**

| **Metabolite** | **Migraine phase effects** | | | |
| --- | --- | --- | --- | --- |
|  | Change from baseline to preictal phase |  | Change from baseline to ictal phase |  |
|  | Estimate (95% CI) | p-value | Estimate (95% CI) | p-value |
| Glutamate | 0.25 (-0.18 – 0.67) | 0.244 | 0.15 (-0.30 – 0.60) | 0.500 |
| Glutamine | 0.20 (-0.29 – 0.68) | 0.422 | 0.24 (-0.24 – 0.72) | 0.316 |
| GABA | 0.46 (0.06 – 0.86) | **0.025** | 0.30 (-0.10 – 0.69) | 0.136 |
| GSH | -0.10 (-0.28 – 0.07) | 0.236 | -0.04 (-0.21 – 0.13) | 0.652 |
| tNAA | 0.19 (-0.30 – 0.68) | 0.445 | 0.17 (-0.31 – 0.66) | 0.477 |
| tCr | 0.06 (-0.27 – 0.38) | 0.729 | 0.10 (-0.21 – 0.42) | 0.515 |
| Ins | -0.02 (-0.35 – 0.32) | 0.917 | 0.06 (-0.28 – 0.39) | 0.736 |
| tCho | 0.03 (-0.04 – 0.09) | 0.364 | 0.007 (-0.06 – 0.07) | 0.826 |
| Aspartate | 0.12 (-0.48– 0.71) | 0.690 | -0.22 (-0.79 – 0.35) | 0.440 |
| PE | 0.22 (-0.04 – 0.49) | 0.097 | 0.16 (-0.08 – 0.40) | 0.177 |

Values are expressed as mean mmol/L and 95% confidence intervals. p-values < 0.05 in bold. GSH = glutathione, Ins = myo-inositol, PE=phosphoethanolamine, tCr= total creatine, tCho = total choline, tNAA = total *N*-acetylaspartate.
